# 布格替尼治疗*ALK*阳性NSCLC患者期间相关早发性肺事件及其管理策略

**DOI:** 10.3779/j.issn.1009-3419.2023.101.12

**Published:** 2023-04-20

**Authors:** YANG Mingyi, LUO Weichi, ZHOU Qing

**Affiliations:** ^1^510080 广州，华南理工大学医学院（杨明意，周清）; ^1^School of Medicine South China University of Technology, Guangzhou 510080, China; ^2^510080 广州，南方医科大学附属广东省人民医院，广东省医学科学院，广东省肺癌研究所（杨明意，罗伟池，周清）; ^2^Guangdong Lung Cancer Institute, Guangdong Provincial People's Hospital, Southern Medical University, Guangdong Academy of Medical Sciences, Guangzhou 510080, China

**Keywords:** 肺肿瘤, 间变性淋巴瘤激酶, 早发性肺事件, 布格替尼, Lung neoplasms, Anaplastic lymphoma kinase, Early-onset pulmonary events, Brigatinib

## Abstract

间变性淋巴瘤激酶（anaplastic lymphoma kinase, ALK）是一种受体酪氨酸激酶，可在非小细胞肺癌（non-small cell lung cancer, NSCLC）中发生重排，导致ALK激酶域信号传导失调。布格替尼是一种强效ALK酪氨酸激酶抑制剂（tyrosine kinase inhibitors, TKIs），于2022年3月在中国获批上市，适应证为ALK重排阳性局部晚期或转移性NSCLC。临床研究显示，与克唑替尼相比，布格替尼显著提升了患者生存和颅内疗效，改善了生活质量，且总体安全性良好，成为ALK阳性NSCLC的治疗优选，同时也为患者带来更多选择。肺部毒性反应是TKIs类药物的不良反应之一，虽然发生率低，但需要临床医生予以重视。布格替尼治疗期间报告的肺毒性反应呈现出独特的临床表现，如早发性（中位发生时间2 d）、发生率与起始剂量相关、患者可快速耐受、症状可逆等。鉴于此，在递交审批过程中提出并确立了早发性肺事件（early-onset pulmonary events, EOPEs）这一概念。本文着重对布格替尼相关的EOPEs临床表现、潜在发病机制、临床管理策略进行论述，为临床医生提供循证医学依据，以支持更好的临床决策。

## Abstract (en)

Anaplastic lymphoma kinase (ALK) is a receptor tyrosine kinase and its rearrangements occur in non-small cell lung cancer (NSCLC), resulting in signal dysregulation in kinase domain. As a new generation of potent ALK tyrosine kinase inhibitors (TKIs), Brigatinib was approved in China in March 2022 as a treatment for locally advanced or metastatic NSCLC patients with ALK rearrangement positive. Brigatinib significantly improved the survival, cranial efficacy and quality of life compared to Crizotinib in clinical trials. Brigatinib is generally well tolerated. Brigatinib has been one of the preferred treatments and an addition of options in ALK-rearranged NSCLC. Pulmonary toxicity is one of the adverse effects observed during the treatment of TKIs and deserves the intense attention of clinicians, despite of its low incidence rate. Pulmonary toxicity reported during the treatment of Brigatinib has shown distinct clinical presentations, such as early-onset (median time to onset, 2 days) and rapid tolerance and reversibility of symptoms. In view of this, the concept of early-onset pulmonary events (EOPEs) was proposed and established during the submission for regulatory review and approval. We focused on clinical characteristics, potential mechanism of etiology, and management strategies of EOPEs to provide clinicians evidence for better clinical decision support.

## 1 引言

间变性淋巴瘤激酶（anaplastic lymphoma kinase, ALK）是一种受体酪氨酸激酶，可在非小细胞肺癌（non-small cell lung cancer, NSCLC）中发生重排，导致ALK激酶域信号传导失调^[1]^。ALK重排阳性亚型占NSCLC总人群的3%-7%^[2]^，中国NSCLC人群的5.6%-9.1%^[3⇓-5]^。最常见的融合伴侣为棘皮动物微管相关样蛋白4（echinoderm microtubule associated protein like 4, EML4），其他有KIF5B、TFG、HIP1等^[1,6]^。ALK重排主要见于年轻、非吸烟患者、腺癌组织学类型，但这些典型的临床病理特征并非诊断特异的^[6]^。美国国立综合癌症网络（National Comprehensive Cancer Network, NCCN）指南推荐，无论年龄或吸烟状态如何，推荐转移性非鳞癌NSCLC患者均进行ALK检测^[1]^。

ALK靶向治疗目前是ALK重排阳性NSCLC的标准治疗。ALK酪氨酸激酶抑制剂（tyrosine kinase inhibitors, TKIs）显著改善了ALK重排阳性NSCLC的预后。布格替尼为一种强效ALK-TKIs，与克唑替尼相比，布格替尼显著提升了患者的生存（ALTA-1L研究随访40.4个月时中位无进展生存期为24.0个月 vs 克唑替尼11.1个月，风险比=0.48，P<0.0001）和颅内疗效（基线期伴任意脑转移亚组4年总生存71% vs 克唑替尼44%，P=0.020），改善了生活质量^[7]^，并对ALK继发性耐药突变显示了广泛临床前活性^[8]^。2017年4月美国食品药品监督管理局（Food and Drug Administration, FDA）加速批准布格替尼用于治疗克唑替尼治疗进展或不能耐受的ALK阳性NSCLC、2020年新增ALK阳性NSCLC一线适应证，2022年3月中国国家药品监督管理局（National Medical Products Administration, NMPA）批准布格替尼作为ALK阳性局部晚期或转移性NSCLC患者治疗。布格替尼也被NCCN指南推荐为一线优选治疗方案^[1]^。

布格替尼总体安全性良好，最常见的（≥25%）不良反应为腹泻、血液磷酸肌酸激酶升高、咳嗽、恶心、高血压等^[9]^。值得注意的是，布格替尼治疗期间报告了一系列早发性肺事件（early-onset pulmonary events, EOPEs），这未曾在既往ALK-TKIs注册临床研究中观察到。这种肺事件本质上属于间质性肺疾病（interstitial lung disease, ILD）或非感染性肺炎。研究者发现布格替尼研究中的肺事件呈现出独特的自然病程，如发生时间早、与起始剂量相关、大多数患者在继续治疗的情况下能够快速耐受且症状可逆等^[10]^。鉴于其早发性等特点，同时区别于传统ALK-TKIs引起的常见ILD或非感染性肺炎，在布格替尼新药申请和生物制品许可申请递交审批过程中提出并确立了EOPEs这一概念^[11]^。EOPEs虽然发病率很低，但可能有致命性，需获得临床医生的充分认知与重视。本文将探讨布格替尼相关EOPEs的诊断标准、临床表现、潜在发病机制、剂量调整及临床管理策略，以此为医生提供循证依据来支持临床决策。

## 2 布格替尼相关的EOPEs

### 2.1 EOPEs的定义与诊断标准

根据药品评价与研究中心多学科回顾报告，EOPEs的定义与诊断标准如下^[11]^：（1）具有时间相关性（定义为体征/症状发生于开始治疗、暂停后继续治疗或剂量增加后≤7 d）；（2）非感染性肺炎样证据[如，低氧或呼吸困难；伴影像学或病理学支持证据，如计算机断层扫描（computed tomography, CT）/X线磨玻璃影，或组织病理学弥漫性肺泡损伤]；确定不可能存在其他病因（如感染、肿瘤等）；（3）另外，若明确证据显示剂量暂停后肺部事件缓解，或重新开始治疗后肺部事件复发，则可辅助确诊。

基于上述标准，若符合（1）-（3）的患者为EOPEs确诊病例，若符合（1）-（2）为疑似病例，若不符合（1）或（2）则排除EOPEs。

类似地，对于发生于7 d以上且符合（2）或（2）+（3）的病例被视为非感染性肺炎[符合（2）为疑似病例，符合（2）+（3）为确诊病例]^[11]^。

需指出，EOPEs的定义是在对布格替尼研究中符合纳入标准的病例进行分析后确立的。布格替尼临床研究方案最初将早期肺事件的发生时间定义为开始治疗后≤14 d，研究结果也按此标准进行报告。在药物审批期间，药品评价与研究中心将≤14 d时间窗内发生的肺事件全部纳入分析，在最终确定的EOPEs定义中将发生时限界定为≤7 d^[11]^。但既往已发表的综述^[12]^以及本文仍遵循现有临床研究报告^[13⇓⇓-16]^，在总结EOPEs发生率数据和回顾个体病例时，将发生于7 d-14 d的肺事件保守地视为EOPEs。

### 2.2 EOPEs的临床特征

布格替尼相关的EOPEs具有早发性特点，中位发生时间为48 h（范围：1 d-9 d），临床表现为呼吸困难、缺氧、咳嗽，具有非感染性肺炎样影像学改变，但无明确的感染原因，大多数患者在继续治疗的情况下可快速耐受且症状可逆^[7,13⇓⇓-16]^。布格替尼一线治疗研究中EOPEs发生率为2.9%（4/136），与克唑替尼经治（包括交叉换组）人群合并后分析显示，其总发生率为4.3%（20/470）（表1）^[7,9,12⇓⇓⇓⇓-17]^。

**表1:** 布格替尼I期-III期研究中EOPEs发生情况总结

Study	Phase	Patients	Brigatinib treatment	n	EOPEs of any grade, grades 3-4	EOPEs-related treatment interruption or discontinuation
ALTA-1L (Study 301)*^ [7,9,13,17]^	3	ALK-TKIs naive; ≤1 line previous systemic treatment for advanced NSCLC	90 mg→180 mg qd	136 (Brigatinib arm); 65 (Crizotinib arm crossed-over to Brigatinib arm)	Brigatinib arm: 2.9% (4/136), 2.2% (3/136); Patients crossed over to Brigatinib arm: 1.5% (1/65), 1.5% (1/65)	Brigatinib was permanently discontinued in all 5 patients after the occurrence of pulmonary events per protocol. Of which, three of four patients in Brigatinib arm and 1 patient crossed over to Brigatinib arm discontinued Brigatinib because the severity of the initial event was grade 3 or 4; for the remaining patient who had initially grade 2 pneumonitis on day 8, the event improved to grade 1 following interruption of Brigatinib, then developed to grade 3 five days after Brigatinib was restarted at 60 mg qd, so Brigatinib was permanently discontinued.
ALTA（Study 201）^#[9,14,15,17]^	2	Disease progression during previous treatment with Crizotinib	Arm A: 90 mg qd; Arm B: 90 mg→180 mg qd	219 (treatment population: Arm A 109+ Arm B 110)	6.4% (14/219)^†^, 2.7% (6/219), (all occurred at 90 mg qd)	Six patients resumed Brigatinib after dose interruption; one patient continued Brigatinib after dose reduction to 60 mg without needing interruption; seven patients discontinued Brigatinib.
NCT01449461（Study 101）^[9,16,17]^	1/2	ALK-TKIs naive or Crizotinib-treated	30 mg-300 mg qd; 60 mg-120 mg bid	50 (90 mg qd; total of all dose groups 137)	2% (1/50), 2% (1/50)	One patient experienced grade 3 event on day 3, resolved after drug withdrawn without rechallenge.
Summary					Any grade: 4.3% (20/470)	

*In ALTA-1L (study 301), EOPEs referred to ILD or pneumonitis, both of which were reported at the 90 mg qd stage (ie. before increasing to 180 mg qd), and occurred within 3-8 days; ^#^In ALTA (Study 201), EOPEs referred to ILD or pneumonitis, pneumonia and dyspnea, which all occurred at 90 mg qd (for Arm B, that is, before increasing to 180 mg qd), and the onset was within 1-9 days (median 2 days); ^†^Includes a grade 5 event. One patient with grade 5 pneumonia after taking Brigatinib 90 mg qd for 7 days^[15]^. Although the cause of death was ascribed to lung cancer and infection, an association with EOPEs could not be ruled out^[8,12]^. EOPEs: early-onset pulmonary events; qd: once a day. ILD: interstitial lung disease; ALK-TKIs: anaplastic lymphoma kinase-tyrosine kinase inhibitors; NSCLC: non-small cell lung cancer.

### 2.3 EOPEs的潜在发生机制

EOPEs为ILD或非感染性肺炎的特殊（早发性）形式，推测多种病理生理机制可能参与其发生和发展，主要包括：（1）克唑替尼洗脱的影响：克唑替尼为CYP3A4的时间依赖性抑制剂^[18]^，而布格替尼主要经CYP2C8与CYP3A4代谢^[9,17]^。从药物相互作用的角度，若克唑替尼洗脱期较短，可能导致布格替尼代谢清除减少，且起始剂量后的药物暴露增加，进而使不良反应风险增加。然而，针对健康志愿者与癌症患者（其中201例为ALK阳性NSCLC）的群体药代动力学分析显示，克唑替尼洗脱期长短并非影响布格替尼首次给药后暴露水平的显著因素^[19]^。值得注意的是，布格替尼≥二线治疗研究要求既往克唑替尼与布格替尼首次给药间隔>3 d^[15]^，提示前述分析纳入的克唑替尼经治患者中克唑替尼对布格替尼暴露的影响可能并无临床意义。这还需临床研究与实践的进一步探索。（2）对血管生理功能的靶外效应：布格替尼是唯一证实具有抗表皮生长因子受体（epidermal growth factor receptor, EGFR）关键突变的临床活性，且与高血压相关的ALK-TKIs，提示对血管生理有一定的靶外效应^[10]^。EGFR表达于II型肺细胞，参与肺泡壁的修复^[20,21]^。对EGFR的抑制可直接损伤肺泡毛细血管内皮和/或肺细胞，潜在地加重其他原因引起的肺损伤程度。而细胞因子释放和炎症细胞募集进一步诱导内皮功能障碍和肺水肿^[20,21]^。（3）基础肺功能：基线期肺功能较差的患者，可能更容易表现出短暂药物诱导性呼吸功能下降相关的症状，或与EOPEs的发病有潜在的相关性。TKIs相关的临床研究通常将ILD或药物诱导的非感染性肺炎既往史作为排除标准，布格替尼关键研究也同样排除了这类患者。因此，仍需要进一步的研究评估基础肺部病变对EOPEs发生发展的影响^[10,13⇓⇓-16]^。

### 2.4 EOPEs的风险因素

目前讨论充分的布格替尼相关的EOPEs风险因素包括起始剂量较高、老年患者（≥65岁）以及与既往克唑替尼治疗的给药间隔<7 d^[10,12,17]^。

#### 2.4.1 起始剂量

布格替尼起始剂量越大，EOPEs发生率越高。I期/II期研究中，各个剂量组综合来看，8%的患者在开始治疗后，或暂停继以重新开始治疗后的7 d内出现肺事件，中位48 h，而且发生率随着起始剂量的增加而升高：90 mg qd、120 mg qd、180 mg qd、240 mg qd、300 mg qd组分别为2%、9%、14%、10%、50%^[16]^。I期/II期研究最初确定的II期研究推荐剂量（recommended phase 2 dose, RP2D）为180 mg qd，因为该剂量下无剂量限制性毒性发生，同时兼顾了初步的临床活性和药代动力学特征。然而，鉴于180 mg qd起始剂量组EOPEs累积发生率高达14%，进一步探索了2种RP2D——90 mg qd和90 mg→180 mg给药方案^[16]^。研究结果发现，90 mg→180 mg方案证实能够在保持高药物暴露带来的长期无进展生存获益和颅内有效性及总生存获益的同时，有效降低EOPEs发生风险^[16]^。I期/II期研究中，90 mg→180 mg组32例患者（28例为ALK重排阳性NSCLC）在180 mg剂量阶段均未发生EOPEs^[16]^。实际上，若临床意义上的EOPEs发生率随着起始剂量的增加而升高，那么采用较低的起始剂量，直至出现耐受后再增加剂量，这可能允许患者更安全地增加剂量并度过亚临床EOPEs风险期的转变过程^[10]^。这一剂量递增方案的安全性得到后续II期和III期研究的证实。在ALTA和ALTA-1L研究中，观察到的全部EOPEs均发生于90 mg qd阶段，中位发生时间为48 h，增至180 mg qd后无新发病例（表1）^[7,13⇓-15]^。Gupta等^[19]^发表的暴露-反应关系分析提供了临床药理学方面的支持，证明布格替尼90 mg→180 mg剂量递增方案具有最佳的获益-风险比。该发现推动了布格替尼RP2D与最终批准的剂量与减量方案的确定（表2）。

**表2:** 推荐的布格替尼常规剂量与减量方案

Dosage	Dosage reduction		
	First	Second	Third
90 mg qd (the first 7 days）	60 mg qd	Permanently discontinue	Not applicable
180 mg qd	120 mg qd	90 mg qd	60 mg qd

Once reduced for adverse reactions, do not subsequently increase the dosage of Brigatinib. Permanently discontinue Brigatinib if patients are unable to tolerate 60 mg once daily dose.

#### 2.4.2 年龄

EOPEs在≥65岁患者中发生率高于<65岁患者（10.1% vs 3.1%）^[17]^。EOPEs与年龄增长之间的相关性可能归于老年患者肺功能减退，而基线呼吸功能较差的患者可能更容易表现出常见的短暂药物诱导性呼吸功能下降引起的症状^[10]^。然而，布格替尼研究人群的已知既存肺疾病数据不够充分，不足以作为独立参数纳入风险因素分析或进行与年龄之间的交互作用分析^[10,12,17]^。尽管如此，老年患者用药期间仍应接受更密切的观察与评估。

#### 2.4.3 与既往克唑替尼治疗的给药间隔

EOPEs在既往克唑替尼末次给药与布格替尼首次给药的时间间隔<7 d的患者中发生率高于≥7 d（9.2% vs 3.6%）^[17]^。不过，前文发生机制中已经提到，目前尚无证据支持克唑替尼洗脱期长短与布格替尼首次给药后暴露水平之间存在显著的相关性。考虑到克唑替尼经治者为布格替尼治疗的候选人群，临床实践中仍应注意给药间隔的评估。

值得讨论的是，欧洲药品管理局（EMA）说明书指出年龄和给药间隔为EOPEs的独立风险因素^[17]^，但二者在Ng等^[12]^的多变量回归分析中并无统计学意义。该分析认为基线期东部肿瘤协作组体能状态评分（Eastern Cooperative Oncology Group performance status, ECOG PS）[0-1 vs 2; OR=0.28; 95%CI: 0.09-0.92; P=0.035]和既往治疗方案数（每增加1个方案；OR=1.26；95%CI：1.03-1.52；P=0.022）与EOPEs显著相关。需指出，Ng等^[12]^汇总分析纳入的I期/II期研究EOPEs数据（8%, 11/137）涵盖了90 mg-300 mg qd共5个剂量组的11例患者，其中仅1例来自90 mg qd组，而这11例在分析的全部EOPEs病例中（8%, 11/137; 6%, 14/219; 3%, 4/136）占38%（11/29）。因此，该汇总分析结果可能不适合作为接受布格替尼推荐剂量方案的患者风险评估。

### 2.5 EOPEs的预测因子

Ng等^[22]^的前瞻性、观察性队列研究首次评估了患者开始布格替尼治疗后的肺功能变化，以探索能够预测EOPEs发生的指标。

#### 2.5.1 一氧化碳扩散能力（diffusing capacity for carbon monoxide, DLCO）

有报告^[22,23]^指出，DLCO是间质病变以及药物诱导肺毒性反应的敏感指标，DLCO数值低提示预后较差。在接受布格替尼治疗后的8 d内，大部分患者经历了DLCO小幅度下降，其中30%（3/10）达到基于肺功能检测的EOPEs定义标准（根据该研究方案DLCO相对于基线值下降≥20%）。全部患者在继续用药和剂量增加的情况下，第15天DLCO改善。最重要的是，上述患者均未表现出与DLCO变化相一致的明显的肺部症状^[22]^。仍需深入研究以明确DLCO下降对布格替尼治疗而言是否具有特异性，以及DLCO下降是否伴有相应的影像学改变。

#### 2.5.2 治疗前活化中性粒细胞水平

ILD相关研究观察到哮喘、慢性阻塞性肺疾病以及支气管扩张病例中中性粒细胞活性升高与肺功能下降相关^[22]^。与此一致，布格替尼前瞻性观察性研究中，质谱流式细胞技术（cytometry by time of flight, CyTOF）分析显示，肺功能检测确诊的EOPEs患者在基线期和第8天具有显著较高的活化中性粒细胞水平（CD15^+^ pERK^hi^）^[22]^。有必要在更大规模人群中评估治疗前活化中性粒细胞水平能否作为EOPEs发生的生物标志物。

## 3 布格替尼相关EOPEs的管理

### 3.1 诊断与评估

接受布格替尼治疗的患者若疑诊EOPEs，应进行基线期评估，包括临床检查、放射影像学检查以及与感染性疾病的鉴别诊断等^[24]^（图1）。

**图 1:**
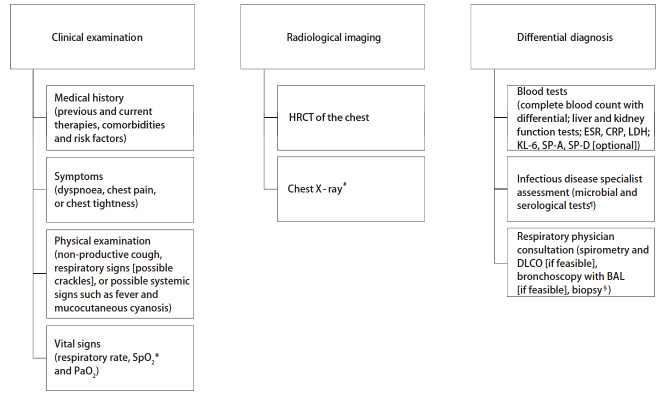
EOPEs诊断与评估流程：临床检查、影像学评估以及鉴别诊断

### 3.2 分级

EOPEs分级标准可参考ILD或非感染性肺炎美国国家癌症研究所不良事件通用术语标准（National Cancer Institute Common Terminology Criteria for Adverse Events, NCI CTCAE）5.0版（表3）^[25]^。1级EOPEs无症状，其确诊取决于影像学证据；2级-4级EOPEs不一定出现全部常见的症状和体征；1级-4级EOPEs的诊断均为排除性的^[24]^。就严重程度而言，1级-2级相当于轻中度，3级为重度，4级为极重度。

**表3:** 间质性肺疾病/非感染性肺炎分级标准

CACTE term	Grade 1	Grade 2	Grade 3	Grade 4	Grade 5
Pneumonitis (inflammation focally or diffusely affecting the lung parenchyma)	Asymptomatic; clinical or diagnostic observations only; intervention not indicated	Symptomatic; medical intervention indicated; limiting instrumental ADL	Severe symptoms; limiting self-care ADL; oxygen indicated	Life-threatening respiratory compromise; urgent intervention indicated (e.g., tracheotomy or intubation)	Death

According to Common Terminology Criteria for Adverse Events (CTCAE) version 5.0 published by National Cancer Institute (NCI)^[25]^. ADL: activity of daily living.

### 3.3 诊治与随访

布格替尼治疗期间若患者发生EOPEs，应参考布格替尼说明书中对ILD或非感染性肺炎的剂量调整建议^[9,17,26]^，以及癌症药物诱导的ILD诊疗方面的专家观点^[24]^。

开始治疗前应告知患者，布格替尼或可发生罕见的EOPEs。在患者教育中应强调密切留意症状变化并及时报告的重要性。一旦发生EOPEs，应按照分级进行管理[1级-2级EOPEs需根据说明书要求进行剂量调整及对症处理，3级-4级EOPEs则需永久停用布格替尼（图2）、制定支持治疗以及随访策略（图2，表2，表3）]^[9,17,24⇓-26]^。不一定需要全部完成前述诊断流程才开始治疗，尤其对于临床恶化的病例。3级EOPEs症状导致生活质量下降，患者可能需要吸氧，无论影像学严重程度如何；4级患者需住院以及考虑机械通气的必要。1级-4级EOPEs需要不同强度的短期类固醇治疗，缺氧症状完全缓解后，类固醇需逐渐减量（推荐的减量期：1级至少4周，2级4周-6周，3级-4级8周-12周）。早期中断类固醇治疗或减量过快可增加EOPEs重新激活的风险或导致现有EOPEs恶化^[24]^。包括肿瘤科、放射科、呼吸科、药理科以及传染病科在内的多学科团队应全程参与确诊评估、治疗管理以及随访监测。而目前已获NMPA批准的其他ALK-TKIs，包括克唑替尼、塞瑞替尼、阿来替尼、恩沙替尼和洛拉替尼，在治疗过程中一旦出现ILD或非感染性肺炎，无论级别，均应永久性终止治疗（表4）^[27⇓⇓⇓-31]^。

**图 2:**
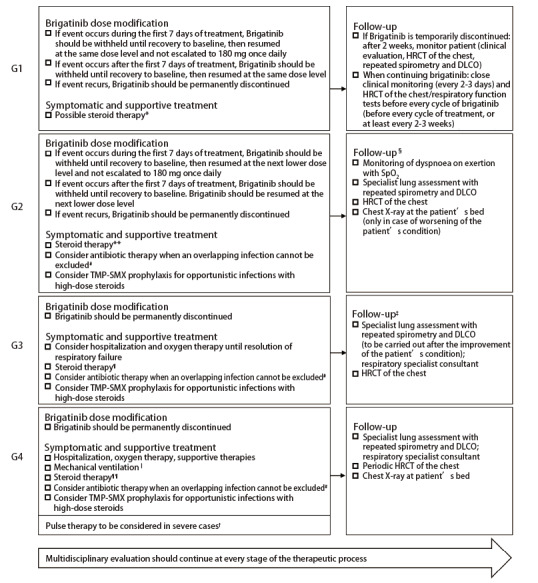
1级-4级EOPEs管理与随访策略：布格替尼剂量调整、对症支持治疗以及随访

**表4:** 其他ALK-TKIs针对ILD/非感染性肺炎的剂量调整

ALK-TKIs	Dose adjustment principle in case of ILD or pneumonitis
Crizotinib	Any grade drug-related ILD/pneumonitis: permanently discontinue^[27]^
Alectinib	Any grade treatment-related ILD/pneumonitis: permanently discontinue^[28]^
Ceritinib	Any grade treatment-related ILD/pneumonitis: permanently discontinue^[29]^
Ensartinib	Any grade drug-related ILD/pneumonitis: permanently discontinue^[30]^
Lorlatinib	Any grade treatment-related ILD/pneumonitis: permanently discontinue^[31]^

## 4 总结

EOPEs为布格替尼治疗相关的罕见肺部不良反应，临床表现总体上符合ILD或非感染性肺炎，且具有独特的疾病过程，包括早发性（中位发生时间2 d）、与起始剂量相关、大多数患者在继续治疗的情况下能够快速耐受、症状可逆等特点，这些均不同于传统ALK-TKIs引起的ILD或非感染性肺炎。ILD或非感染性肺炎是ALK-TKIs在NSCLC人群中的不良反应之一，在晚期ALK阳性NSCLC患者中，ALK-TKIs相关ILD或非感染性肺炎的任何级别发生率为2.14%（95%CI: 1.37%-3.34%），≥3级为1.33%（95%CI: 0.80%-2.21%）^[32]^。就发生时间而言，从开始克唑替尼治疗到发生ILD或非感染性肺炎的中位时间为23 d-1个月^[33]^，恩沙替尼研究显示的发生时间为用药后2个月^[30]^，其他ALK-TKIs尚无至事件发生时间的汇总分析。除布格替尼以外，已获批ALK-TKIs的临床试验并未报告过EOPEs。然而，文献检索发现有一些ALK-TKIs有发生时间与EOPEs相似的ILD或非感染性肺炎的个案报告。例如2013年-2018年共有6例在开始克唑替尼一线至三线治疗2周内出现ILD或非感染性肺炎的临床病例报告，其中2例基线期既存非特异性ILD或恶性胸腔积液^[34⇓⇓⇓-38]^。2例患者的事件分别发生于第3、7天^[35,37]^，其余为第9-13天^[34,36,38]^；4例死亡^[34,35,37]^，其余2例在ILD或非感染性肺炎缓解后因患者意愿或扩展供药项目许可继续使用克唑替尼或转换为布格替尼治疗^[36,38]^。另外，Monzonis等^[39]^报告了1例患者在四线布格替尼治疗第7天出现非感染性肺炎，缓解后继续布格替尼治疗5个月未复发，五线治疗给予洛拉替尼1 d后出现复发。以上发生于开始治疗后2周内的事件均为在临床实践中观察到的个别案例，相对更远期的肺毒性反应而言十分罕见。不过，最近发表的WX-0593（Iruplinalkib）I期研究中观察到2例用药后48 h内因肺事件死亡的病例（其中1例死因为感染性肺炎，1例死因为呼吸衰竭），研究者判断这可能是与WX-0593相关的肺事件，因此修改了研究方案，纳入低剂量导入期，之后未再观察到早期肺事件^[40]^。然而，这项研究并未明确EOPEs定义，未来包括WX-0593在内的其他新药是否存在EOPEs有待进一步研究数据揭示。

布格替尼相关EOPEs发生的病理生理机制仍不清楚，现有的数据提示EOPEs的发生机制可能并不等同于常见的ALK-TKIs诱导性ILD或非感染性肺炎。在其他药物的早期肺事件中，有病例报告分析认为，克唑替尼导致的少见的ILD急性形式可能归于药物直接毒性作用，并不涉及免疫源性机制，而后者可能与其经典或常见的ILD非急性形式有关^[37]^。至于药物直接毒性作用是否为布格替尼相关EOPEs的一个驱动因素，尚需更深入的理解和病例再评估。此外，少数病例报告描述了临床上一种ALK-TKIs诱导ILD后继续治疗或转换为其他ALK-TKIs之后肺毒性的复发情况，包括克唑替尼诱导ILD后继续使用克唑替尼^[36]^或转换为布格替尼^[38]^或阿来替尼^[41]^（各1例）、阿来替尼诱导ILD后继续使用阿来替尼^[42]^或转换为洛拉替尼 ^[43]^（各2例），均未见肺毒性反应复发，也有布格替尼诱导EOPEs后转换为洛拉替尼时，EOPEs再次出现的病例（1例）^[39]^。不同ALK-TKIs在诱导肺毒性反应方面是否存在交叉反应性，也仍无定论。

除布格替尼外，其他5种已在国内获批的ALK-TKIs（克唑替尼、阿来替尼、塞瑞替尼、恩沙替尼以及洛拉替尼）^[27⇓⇓⇓-31]^说明书均建议，对于用药过程中出现提示ILD或非感染性肺炎的肺部症状急性发作和/或不明原因加重的患者，应直接就医，以排除是否为ILD或非感染性肺炎；在查找病因期间，应暂停治疗，根据症状与体征考虑吸氧、抗炎和抗生素等对症支持治疗；若确诊ILD，则应永久性终止治疗^[27⇓⇓⇓-31]^。考虑到布格替尼相关EOPEs的独特性，建议1级或2级患者严格按照布格替尼说明书进行恰当的剂量调整、监测临床表现和实验室报告变化、积极采取对症支持治疗等有效管理手段；3级或4级则永久性终止治疗^[9,17,26]^。

布格替尼90 mg→180 mg剂量方案是得到确凿的循证依据支持的给药方式，能够明显降低EOPEs发生率，同时保持高药物暴露带来的长期生存获益和卓越的颅内疗效，具有最佳的获益-风险比。在疑诊或确诊EOPEs的情况下，包括肿瘤科、放射科、呼吸科、药理科以及传染病科在内的多学科团队应全程参与确诊评估、治疗管理以及随访监测。另外，详尽的患者教育以及密切的医患沟通是至关重要的。应充分告知和提醒患者观察自身呼吸状况，及时报告新出现或恶化的症状。完善的预见性治疗管理策略有助于确保患者安全地从布格替尼治疗中得到最大的生存获益。

**利益冲突：**本文作者均声明无利益冲突。
